# Sentinel Node Mapping with Blue Dye Injection after Lumpectomy Specimen Removal: Implications for Fluorescence-Guided Breast Surgery

**DOI:** 10.1245/s10434-024-16604-y

**Published:** 2024-12-14

**Authors:** Heidi S. Santa Cruz, Julia N. Shanno, Alexandra J. Webster, Francys C. Verdial, Michele A. Gadd, Michelle C. Specht, Barbara L. Smith

**Affiliations:** Breast Section, Division of GI and Oncologic Surgery, Massachusetts General Hospital, Boston, MA USA

Neoadjuvant chemotherapy is currently used in 30% of patients, based on tumor subtype^1^. Dual-agent mapping with preoperative injection of radioactive colloid and blue dye is mandatory for sentinel lymph node (SLN) mapping after neoadjuvant therapy for breast cancer^2^. However, isosulfan blue and methylene blue dyes used for node mapping produce strong fluorescence that precludes use of some fluorescent optical imaging agents, including pegulicianine, an FDA-approved agent for breast lumpectomy margin assessment^3,4^ that fluoresces in the same wavelengths as sentinel node blue dyes. With increasing interest in real time, intraoperative lumpectomy margin assessment, flexible options for SLN mapping are needed. We investigated the feasibility and efficacy of injecting blue dye for sentinel lymph node (SLN) mapping after excision of the lumpectomy specimen.

## Methods

After Institutional Review Board approval, we identified female breast cancer patients with and without neoadjuvant therapy who received isosulfan blue dye injection for SLN mapping after lumpectomy excision. All had preoperative subareolar Technetium-99 (Tc-99) sulfur colloid injection. Immediately after lumpectomy specimen excision, 1 mL of isosulfan blue mixed with 4 mL of saline was injected into the lumpectomy cavity wall or into subareolar tissue if the lumpectomy cavity was not between the areola and axilla, followed by a 2-min breast massage. Sentinel lymph node biopsy was completed, and blue nodes and blue perinodal lymphatics were documented. Patient, tumor, and procedure data were collected.

## Results

Forty breast cancer patients, mean age 55 years, received blue dye injection after lumpectomy specimen excision. Twenty-three had received neoadjuvant therapy, and 11 patients had a biopsy-positive node at diagnosis—10 of whom had preoperative radiofrequency tag node localization. Thirty-nine patients had hot axillary nodes after Tc-99 mapping.

Sentinel lymph nodes were identified in all 40 patients (Table 1). Blue nodes or lymphatics were identified in 33 patients (82.5%). Blue nodes and/or lymphatics were identified in 100% (6/6) with subareolar injections and in 90% (27/30) with cavity wall injections and SLN excision through a separate axillary incision. No blue nodes or lymphatics were identified after cavity wall injection and SLN excision through the same incision (0/3), or after deep parenchymal injection remote from the cavity (0/1).

**Table 1:** Tumor data and mapping outcomes with blue dye injection after lumpectomy

	Total (N = 40)
*Indication for lumpectomy*	
IDC ± DCIS	35 (87.5%)
ILC ± DCIS	5 (12.5%)
Sentinel node identification	40 (100%)
Focal gamma probe signal	39 (97.5%)
Blue dye injection location	Blue nodes and/or lymphatics identified
Lumpectomy cavity wall with separate SLN biopsy incision	27/30 (90%)
Lumpectomy cavity wall and no separate SLN biopsy incision	0/3 (0%)
Subareolar	6/6 (100%)
Upper outer quadrant deep parenchymal	0/1 (0%)
Number of sentinel lymph nodes removed, mean (range)	3 (1–7)
Patients with positive SLN pathology	8 (20%)
Number of positive SLNs, mean (range)	1 (1–2)

*IDC* invasive ductal carcinoma; *ILC* invasive lobular carcinoma; *DCIS* ductal carcinoma in situ; *SLN* sentinel lymph nodes

## Discussion

Intraoperative blue dye injection after lumpectomy specimen excision was effective for SLN mapping. Prior studies reported blue nodes or lymphatics in 85–87% of patients with preoperative blue dye injection.^5,6^ We observed blue nodes or lymphatics in 82.5% of patients with blue dye injected after lumpectomy specimen excision. Among patients with subareolar injections or cavity wall injections with SLN excision through a separate incision, blue nodes and/or lymphatics were identified in 92% (33/36). The combination of Tc-99 and blue dye successfully identified SLN in 100% of patients. The short 2-min duration of breast massage required after post-lumpectomy blue dye injection did not significantly impact surgical workflow. Blue dye mapping was unsuccessful only after deep parenchymal blue dye injection or when SLN excision was performed through the lumpectomy incision, which carried blue dye injected in the cavity nonspecifically into axillary tissue.

This post-lumpectomy blue dye injection approach also could be considered intraoperatively in patients who had preoperative node mapping with Tc-99 alone and are found to have a weak or absent isotope signal after lumpectomy.

There is increasing interest in options for real time, intraoperative lumpectomy margin assessment in breast cancer patients. Pegulicianine, and other optical imaging agents for tumor detection during cancer surgery, rely on fluorescent signal for tumor identification and are incompatible with blue dye injection before lumpectomy. Our demonstration of successful node mapping with blue dye injection after lumpectomy allows both use of fluorescence-based margin assessment and successful axillary staging.
