# Correction: Sentinel Node Mapping with Blue Dye Injection after Lumpectomy Specimen Removal: Implications for Fluorescence-Guided Breast Surgery

**DOI:** 10.1245/s10434-025-16937-2

**Published:** 2025-01-20

**Authors:** Heidi S. Santa Cruz, Julia N. Shanno, Alexandra J. Webster, Francys C. Verdial, Michele A. Gadd, Michelle C. Specht, Barbara L. Smith

**Affiliations:** Breast Section, Division of GI and Oncologic Surgery, Massachusetts General Hospital, Boston, MA USA

**Correction to: Ann Surg Oncol** 10.1245/s10434-024-16604-y

The article “Sentinel Node Mapping with Blue Dye Injection after Lumpectomy Specimen Removal: Implications for Fluorescence‑Guided Breast Surgery“, written by Santa Cruz et al., was originally published electronically on the publisher’s internet portal on December 14, 2024, without open access. With the authors’ decision to opt for Open Choice the copyright of the article changed on January 10, 2025 to © The Author(s) 2024 and this article is licensed under a Creative Commons Attribution 4.0 International License, which permits use, sharing, adaptation, distribution and reproduction in any medium or format, as long as you give appropriate credit to the original author(s) and the source, provide a link to the Creative Commons licence, and indicate if changes were made. The images or other third party material in this article are included in the article's Creative Commons licence, unless indicated otherwise in a credit line to the material. If material is not included in the article's Creative Commons licence and your intended use is not permitted by statutory regulation or exceeds the permitted use, you will need to obtain permission directly from the copyright holder. To view a copy of this licence, visit http://creativecommons.org/licenses/by/4.0/.

